# Role of HER2 mutations in refractory metastatic breast cancers: targeted sequencing results in patients with refractory breast cancer

**DOI:** 10.18632/oncotarget.5184

**Published:** 2015-09-11

**Authors:** Yeon Hee Park, Hyun-Tae Shin, Hae Hyun Jung, Yoon-La Choi, TaeJin Ahn, Kyunghee Park, Aeri Lee, In-Gu Do, Ji-Yeon Kim, Jin Seok Ahn, Woong-Yang Park, Young-Hyuck Im

**Affiliations:** ^1^ Division of Hematology-Oncology, Department of Medicine, Samsung Medical Center, Sungkyunkwan University School of Medicine, Seoul, Korea; ^2^ Biomedical Research Institute, Samsung Medical Center, Sungkyunkwan University School of Medicine, Seoul, Korea; ^3^ Samsung Genomic Institute, Samsung Medical Center, Sungkyunkwan University School of Medicine, Seoul, Korea; ^4^ Department of Bio and Brain Engineering, Korea Advanced Institute of Science and Technology, Daejeon, Korea; ^5^ Center of Companion Diagnostics, Innovative Cancer Medicine Institute, Samsung Medical Center, Seoul, Korea

**Keywords:** refractory metastatic breast cancer, next generation sequencing (NGS), targeted sequencing, HER2 mutation, HER pathway

## Abstract

In women with metastatic breast cancer (MBC), introduction of the anti-HER2 (human epidermal growth factor receptor-2) directed therapies including trastuzumab, pertuzumab, lapatinib, and/or trastuzumab-DM1 has markedly improved overall survival. However, not all cases of HER2-positive breast tumours derive similar benefit from HER2-directed therapy, and a significant number of patients experience disease progression because of primary or acquired resistance to anti-HER2-directed therapies. We integrated genomic and clinicopathological analyses in a cohort of patients with refractory breast cancer to anti-HER2 therapies to identify the molecular basis for clinical heterogeneity. To study the molecular basis underlying refractory MBC, we obtained 36 MBC tumours tissues and used next-generation sequencing to investigate the mutational and transcriptional profiles of 83 genes. We focused on HER2 mutational sites and HER2 pathways to identify the roles of HER2 mutations and the HER2 pathway in the refractoriness to anti-HER2 therapies. Analysis using massively parallel sequencing platform, CancerSCAN™, revealed that HER2 mutations were found in six of 36 patients (16.7%). One patient was ER (estrogen receptor)-positive and HER2-negative and the other five HER2 mutated patients were HER2-positive and HR (hormone receptor)-negative. Most importantly, four of these five patients did not show any durable clinical response to HER2-directed therapies. The HER2 pathway score obtained through transcriptional analyses identified that Growth Receptor Biding protein 2 (GRB2) was the most significantly down regulated gene in the HER2 mutated samples. Detection of HER2 mutations using higher deep DNA sequencing may identify a predictive biomarker of resistance to HER2-directed therapy. Functional validation is warranted.

## INTRODUCTION

Metastatic breast cancer (MBC) is an incurable disease with a 2- to 3-year median overall survival (OS) time [1, 2]. Although marked advances have been made in HER2-targeted therapies [3–6] and strategies to overcome endocrine resistance [7, 8], some patients exhibit refractory MBC, which do not respond to conventional treatments, and are therefore in need of new therapeutic strategies. When developing such strategies, it is important to realize that patients with MBC are a heterogeneous group, and thus the best strategies for treatment may differ between patients.

The results of next-generation sequencing (NGS) approaches in The Cancer Genome Atlas (TCGA) suggest that primary BCs are mutationally heterogeneous [9, 10]. This heterogeneity of MBCs and refractoriness to conventional treatments represent a significant treatment challenge, and there is a pressing need to understand better the biology of these aggressive cancers and to develop more effective therapies. Progress in cancer genomics has raised hopes of more precise identification of patients suitable for targeted therapies tailored to their genotype. However, there remains a feasibility issue before NGS can be used in making decisions about clinical treatment in daily clinical practice, especially for patients with refractory disease who have only limited options for cancer treatments.

The HER pathway plays a critical role in the pathogenesis of breast cancer, especially in patients with HER2 overexpression [11]. HER-2, together with HER-1, HER-3, and HER-4, is member of the human epidermal growth factor receptor family. HER-1, HER-3, and HER-4 can be activated by various ligands, and their activation triggers conformational rearrangement of the receptor molecules to allow homo- or heterodimerization [12]. Although wild-type HER2 overexpression occurs in 20–30% of breast cancers, Bose et al. estimated that about 1.6% of breast cancer patients possess a HER2 mutation [13]. They found that seven HER2 mutations activated the protein, as reflected in its enzyme activity, and downstream HER2 signaling in mouse xenografts. This observation led to a trial of neratinib, a panHER TKI for patients with refractory HER2 positive BC [13].

Considering this role of HER2 mutations as a driver oncogene, we developed a targeted NGS platform, which we call CancerSCAN™, using the 83 most representative genes (Supplemental Table 1). We used the HiSeq 2500 sequencing platform (Illumina, USA) to investigate the mutational profile of tissues from patients with refractory MBC whose disease progressed after conventional treatments. We focused specifically on HER2 mutations. We also performed whole-transcriptome sequencing (RNA-Seq) to evaluate the HER pathway in patients with refractory MBC.

## RESULTS

### Patients' clinicopathological features (Table 1)

From March 2013 to Nov 2014, 36 patients with MBC that was refractory to conventional treatments were enrolled, and fresh frozen tumour tissues were collected from metastatic sites for targeted sequencing, CancerSCAN™ analysis, and RNA sequencing. Thirty-six tumour samples were analyzed; two patients were excluded because of failure of quality control (QC). The median age at diagnosis of the 36 patients was 45 years (range 26–64). Fourteen patients were hormone receptor (HR)-positive (defined as ER and/or PgR positive). Thirteen BC patients were HER2 positive and 11 patients were triple negative. The median number of chemotherapy regimens before biopsy was 5 (range 3–8) (Table 1). The median follow-up duration from the date of biopsy was 15 months (range 5–20). The median OS time from distant metastasis to death (mOS) was 42.7 months (range 10–81). Among 14 HER2-positive patients, median OS of the patients with HER2 mutation was 20.2 months, which was much shorter than those without HER2 mutation (48.6 months).

**Table 1:** Clinicopathologic features of 36 refractory MBC patients

Patients' number	ID	Age	Menopausal status	ER	PR	HER2	Histologic grade	Nuclear grade	TNM stage at initial Diagnosis	Regimen number of prior chemotherapies at biopsy	Biopsy site
1	OS173	47	Pre	0	0	0	2	2	4	4	Skin
2	OS181	35	Pre	0	0	0	3	3	3A	5	Pleura
3	OS188	42	Pre	0	0	0	3	3	1A	6	Lung
4	OS191	49	Pre	0	0	1	1	2	2A	4	Pleura
5	OS200	30	Pre	1	0	0	3	3	1A	4	Pleura
6	OS209	47	Pre	0	0	0	3	3	3C	6	Skin
7	OS226	51	Pre	0	0	1	3	3	3A	7	LN
8	OS228	38	Pre	0	0	0	N/A	N/A	4	5	Skin
9	OS230	45	Pre	0	0	1	N/A	N/A	1A	5	Breast
10	OS235	55	Post	0	0	0	N/A	N/A	4	6	Liver
11	OS240	56	Post	0	0	1	N/A	N/A	4	3	Breast
12	OS244	46	Pre	0	1	1	N/A	N/A	4	4	Pleura
13	OS245	52	Post	0	0	1	3	3	4	5	Breast
14	OS250	38	Pre	1	0	0	N/A	N/A	2A	5	Breast
15	OS251	40	Pre	0	0	1	2	3	3A	6	LN
16	OS255	44	Pre	1	1	0	3	3	2B	7	Liver
17	OS256	58	Post	0	0	1	3	2	0	7	Liver
18	OS257	56	Post	1	1	1	N/A	N/A	4	7	Breast
19	OS259	42	Pre	0	0	0	2	2	3A	8	Breast
20	OS262	64	Post	0	0	0	N/A	N/A	4	4	Breast
21	OS263	63	Post	1	0	0	N/A	N/A	2A	5	Liver
22	OS265	32	Pre	0	0	1	2	2	4	6	Breast
23	OS266	50	Prel	1	1	0	2	2	4	6	Ovary
24	OS287	52	Post	1	0	0	3	3	3C	4	Pleura
25	OS291	42	Pre	1	0	0	N/A	N/A	3C	7	Pleura
26	OS306	41	Pre	1	1	0	2	2	3C	6	Pleura
27	OS314	63	Post	0	0	1	3	3	4	6	Breast
28	OS321	41	Pre	0	0	1	2	2	2A	5	Breast
29	OS324	47	Pre	1	0	0	3	3	3A	5	Liver
30	OS333	26	Pre	0	1	0	2	2	2A	4	Chest wall
31	OS336	33	Pre	0	0	0	3	3	3C	3	Breast
32	OS337	29	Pre	1	1	1	N/A	N/A	4	6	Breast
33	OS349	36	Pre	1	0	0	N/A	N/A	3C	6	LN
34	OS370	37	Pre	1	1	0	3	3	3A	4	Liver
35	OS375	40	Pre	0	0	0	NA	N/A	4	7	Breast
36	OS410	47	Pre	0	0	0	3	3	2A	3	Breast

0; negative, 1; positive, N/A; not available

### NGS using cancerSCAN™

**Figure 1:**
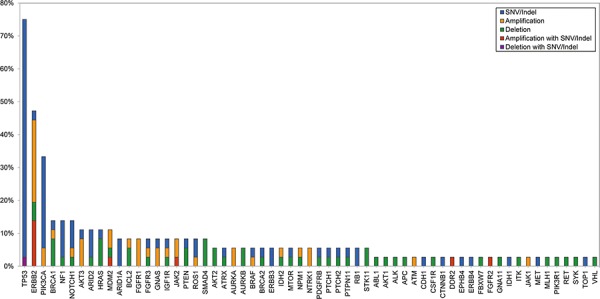
Mutational profile of 36 refractory MBC patients

**Figure 2:**
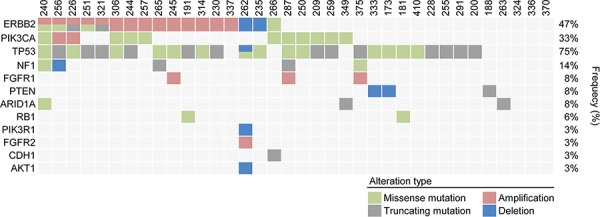
Heat map of patients with genetic alterations of 83 genes among 36 refractory MBC patients

The mean coverage of 36 patients was 941.3× with 99.0% over 100× (Supplemental Table 2). Figure 1 shows the mutational profiles of the 36 patients with refractory MBC. Thirty-five of the 36 patients (97.2%) harboured at least one genetic alteration including copy number variations. A heat-map of mutations of the 83 genes in samples from the 36 patients is shown in Figure 2. A total of 140 genetic alterations were detected in the samples from the 36 patients. Genes in which somatic alterations were detected frequently included TP53 (27 cases, 75%), ERBB2 (17 cases, 47%), PIK3CA (12 cases, 33%), NF1 (five cases, 14%), FGFR (three cases, 8%), PTEN (three cases, 8%), ARID1 (three cases, 8%), RB1 (two cases, 6%), PIK3R1 (one case, 3%), CDH1 (one case, 3%), and AKT1 (one case, 3%), as shown in Figure 1.

### HER2 mutational status of the 36 refractory MBC patients (Table 2, Figure 2)

**Figure 3:**
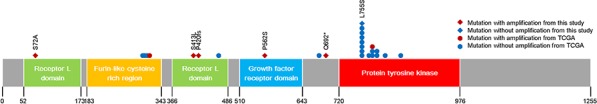
Gene map of ERBB2 mutations

HER2 mutations were found in six of the 36 patients (16.7%, Figure 2). One patient was ER-positive, and the other five patients were HER2-positive. Figure 3 shows the gene maps of the HER2 mutational status of the six patients who harboured HER2 mutations along with the TCGA results. HER2 mutations in three patients were in the receptor ligand domain (RLD). In one patient, the mutation was in the protein tyrosine kinase domain (PTKD), and in the other HER2-negative patient, the mutation was in the growth factor receptor domain (GFRD) (Figure 3). To confirm the mutations in primary tissue, we also genotyped the available archival primary tissue for two patients. We identified one mutation (S413L) with a similar allele frequency to that of metastatic tissue, but none was detected for the other patient.

**Table 2:** Fourteen HER2-positive and one HER2-negative with HER2 mutation patients

Sample.ID	ERBB2	ERBB2	Mutation	Amplification value	GRB2 mRNA	HER2–3 pathway
OS191		Amp. (14.53)	No	14.53	0.674	0.447
OS226	p.P420fs (0.22)	Amp. (13.99)	Yes	13.99	0.144	−0.591
OS230		Amp. (22.38)	No	22.38	0.897	0.178
OS240	S413L (0.01)	Amp. (16.39)	Yes	16.39	0.431	−0.136
OS244		Amp. (12.36)	No	12.36	0.286	−0.037
OS245		Amp. (16.04)	No	16.04	N/A	N/A
OS256	S72A (0.01)	Amp. (25.93)	Yes	25.93	N/A	N/A
OS257		Amp. (30.13)	No	30.13	1.716	−0.470
OS265		Amp. (14.09)	No	14.09	1.189	−0.156
OS306		Amp. (13.8)	No	13.8	N/A	N/A
OS251	P562S (0)	Amp. (20)	yes	20	−0.024	−0.593
OS314		Amp. (9.52)	No	9.52	1.388	−0.485
OS321	Q692X (0.02)	Amp. (5.39)	Yes	5.39	0.541	−0.206
OS337		Amp. (3.99)	No	3.99	0.301	0.040
OS266	L755S (0.19)	None	Yes	2	0.144	−0.591

*NA: not available

### Comparison of HER2 mutations with TCGA data (Supplemental Table 3)

Supplemental Table 3 shows the six HER2 mutations found in our series compared with TCGA data. The VAF percentages were much lower in our series than in the TCGA dataset.

### ERBB2 mutation validation by digital PCR (Table 3)

**Figure 4:**
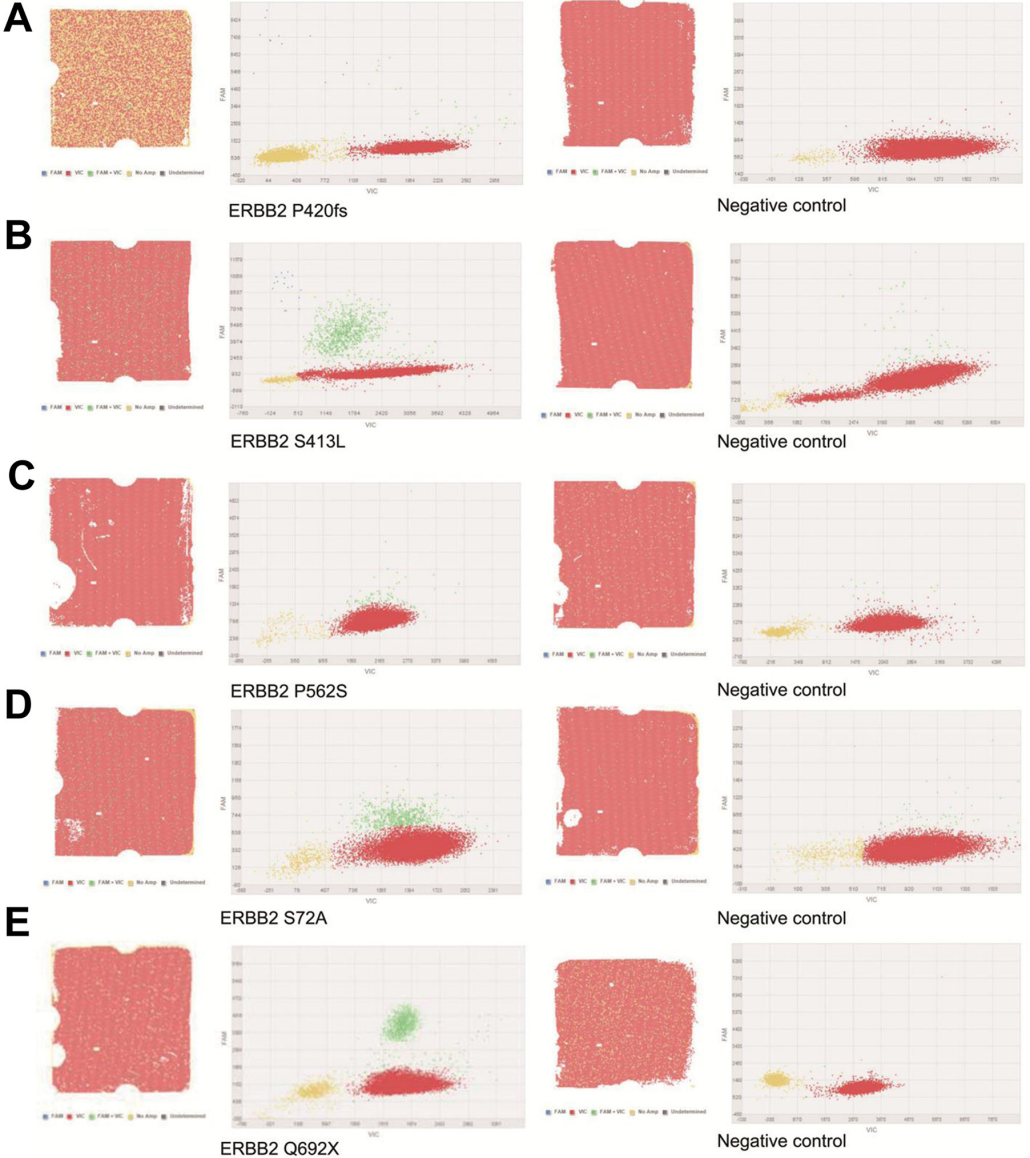
Digital PCR results of the patients who were HER2-positive and HER2 mutation **A.** Patient 226. **B.** Patient 240. **C.** Patient 256. **D.** Patient 251. **E.** Patient 321.

To confirm the low-frequency HER2 mutation, we performed digital PCR assays with negative controls. Among the five samples with HER2 mutations, all mutant alleles in the NGS were detected by digital PCR with similar allele frequency (Figure 4). In each negative control, the signals were much lower in the mutant alleles than in the positive samples (Table 3).

**Table 3:** Digital PCR results of HER2 mutations in five patients with HER2 mutation using NGS

Sample ID	NGS		dPCR* % (FAM/VIC)	
	Amino acid change	VAF% (Alt/Ref)	Negative control	Sample
226	P420fs	0.2172 (16/7365)	0.0103 (3.752/36312)	0.4523 (11.05/2442.6)
240	S413L	1.1954 (96/8668)	0.0670 (35.173/52471)	1.2859 (558.09/45731)
256	S72A	0.7457 (42/5590)	0.0766 (17.506/22848)	0.8343 (190.36/22186)
251	P562S	0.4345 (38/8475)	0.0197 (7.409/37529)	0.2603 (143.46/55095)
321	Q692X	1.6919 (38/2208)	0.0023 (0.601/26319)	1.2574 (433.51/34475)

* dPCR: digital PCR

### HER2 pathway score analysis using RNA-Seq (Figure 5)

**Figure 5:**
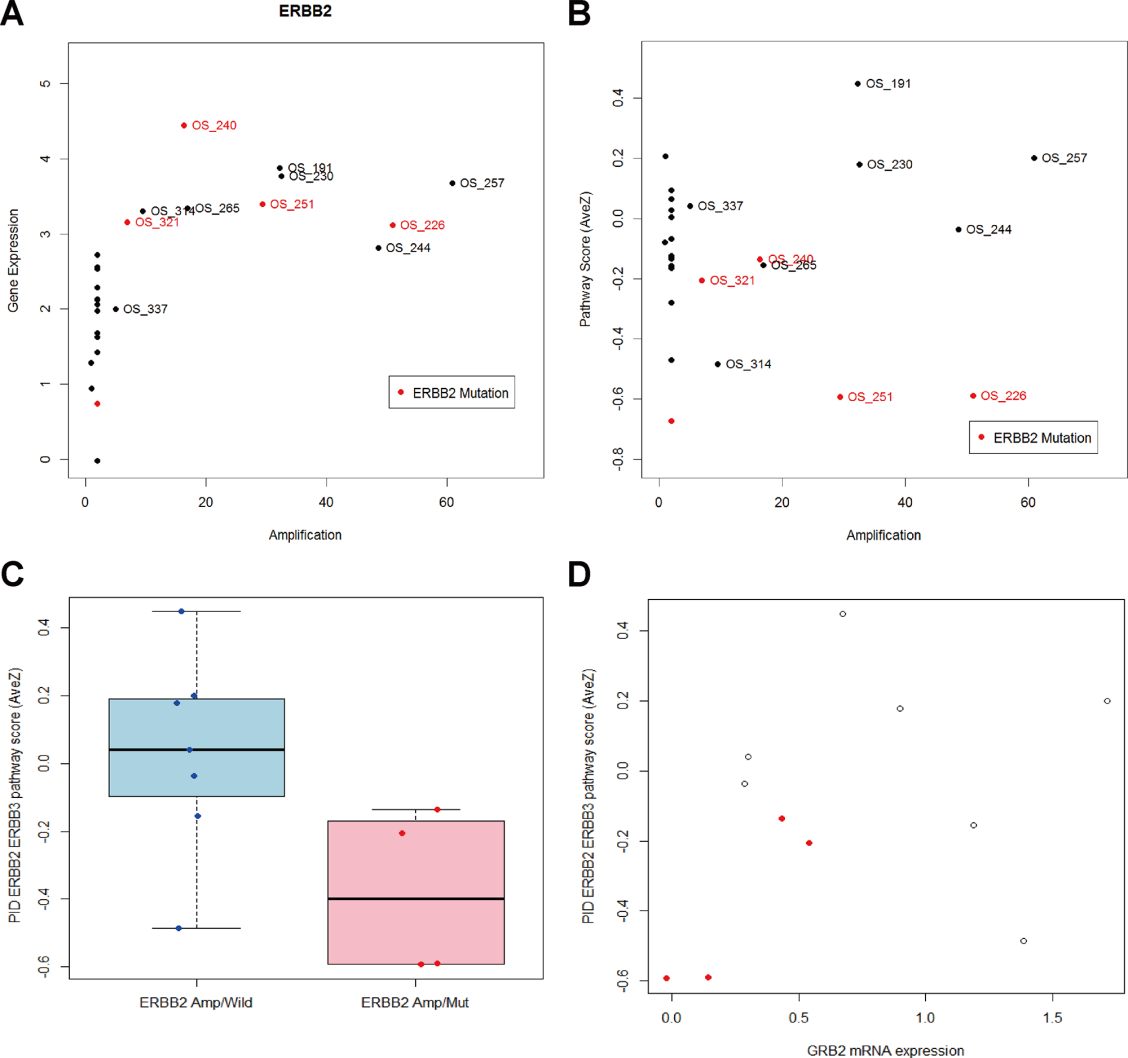
HER2 pathway analysis **A.** ERBB2 copy number and gene expression of twenty seven metastatic breast cancer patients. ERBB2 gene expression is significantly higher in patients with increased copy number. Patients with ERBB2 mutation is red colored. **B.** Averaged mRNA expression of genes in the PID ERBB2 ERBB3 pathway is not associated with ERBB2 amplification. **C.** Among eleven patients with ERBB2 amplification, pathway score is significantly different between patients with ERBB2 mutation and patients without ERBB2 mutation (*P*-value = 0.0413). **D.** ERBB2 amplified and mutated samples yield less expression of GRB2 and pathway level gene expression than ERBB2 amplified and wild.

Standardized mRNA expression values of HER2 show a linear relationship with HER2 copy numbers. The mutation status of the HER2 gene did not alter the mRNA level expression of the HER2 gene in the patients (Figure 5A). Among the patients with an increased HER2 copy number, the average mRNA expression level of the HER2-HER3 pathway differed between the patients with a HER2 mutation and those with the wild-type HER2 (Figure 5B). This was related to down-regulation of several components in the pathway, such as GRB2, PIK3CB, and RAF1 (Supplemental Table 4). Growth Receptor Biding protein 2 (GRB2) was the most significantly down-regulated gene in the HER2-mutated samples. The treatment history of the patients and their GRB2 expression levels are summarized in Tables 2 and 4.

**Table 4:** HER2 mutation status of five HER2-positive MBC patients

Sample.ID	ERBB2	ER	PR	DFS* after surgery followed by adjuvant trastzumab (months)	1^st^-line treatment	PFS^†^ to 1^st^-line treatment	2^nd^-line treatment	PFS to 2^nd^-line treatment	HER2 mutation in archival breast tissue
OS226	p.P420fs (0.22)	Negative	Negative	14	Lapatinib + Capecitabine	4	Gemcitabine + Vinorelbine	9	Not available
OS240	S413L (0.01)	Negative	Negative	Initial stage IV	Trastuzumab + Paclitaxel	38	Lapatinib + capecitabine	6	S413L (0.01)
OS256	S72A (0.01)	Negative	Negative	9	T-DM1	12	Trastuzumab + Docetaxel	1	Not available
OS251	P562S (0)	Negative	Negative	18	Trastuzumab + Paclitaxel	12	Lapatinib + Capecitabine	2	P562S (0)
OS321	Q692X (0.02)	Negative	Negative	15	Trastuzumab + Docetaxel	8	Lapatinib + Capecitabine	1.5	Q692X (0.03)

* DFS: disease free survival

† PFS: progression free survival

### Clinical and mutational characteristics of the six patients with HER2 mutations

To evaluate the role of HER2 mutations in HER2-positive refractory MBC, clinical outcomes and the disease course of the five HER2-positive patients with HER2 mutations were investigated. As shown in Figures 1 and 2 and Table 2, these five patients had HER2-positive BC with HER2 amplification as well as HER2 mutations. However, these mutations did not involve the same site: three were missense and two were truncation mutations, and there was no mutation in the PTKD (Figure 3). Interestingly, no patient was both ER-positive and HER2-positive. All five patients with HER2 mutations in the HER2-amplified BC were HR-negative. Most importantly, four of the five patients did not show any durable clinical response to trastuzumab- and/or lapatinib- containing chemotherapies for more than 12 months (Table 4). They were HER2-positive patients with MBC whose disease was refractory to targeted therapies including trastuzumab-DM1.

## DISCUSSION

Our genomic study results show a higher incidence (16.7%) of HER2 mutations in patients with refractory MBCs than in other series, which is merely 1.6% [13, 16]. This difference may relate to this population of refractory patients or to the deep sequencing method used. Most importantly, our study suggests that HER2 mutations in patients with HER2-amplified BC may contribute to resistance to anti-HER2-directed therapy with chemotherapies, even though only low frequencies of the mutations were found (Table 2), which imply HER2 mutation could be one of the predictive markers of HER2 directed therapy and new resistance mechanism of HER2 pathway in breast cancer. Furthermore, this finding was supported by GRB2 downregulation, which is down-stream of HER2 pathway even though it did not validated CancerSCAN™ (GRB2 is not included in CancerSCAN™ panel). Lim et al., reported down-regulation of GRB2 in heregulin-stimulated-HER2-overexpressing breast cancer cells that lead to reduced proliferation through inactivation of the Akt pathway [14]. It is unclear whether the down-regulation of GRB2 is a consequence of HER2 mutation. It is also possible that the down-regulation of GRB2 might be caused by drug exposure. Decreased expression of GRB2 in lapatinib-treated BC cell lines has been reported [15]. As most (or all) of the HER2-amplified patients in our study had been exposed to HER2-targeting drugs, it is also possible that down-regulation of the GRB2 gene and HER2 pathways might be caused by the treatment, coincidently overlapped with the mutational status of the patient sample. Further functional study of these mutations using an *in-vitro* model may reveal this relationship. Insufficient amount of GBR2 product makes it hard to deliver an HER2-triggered oncogenic signal, therefore patients may be less dependent on HER2-targeted drugs. In spite of this limitation, we decided to try to improve the accuracy of the method to identify HER2 mutations. To exclude false-positive results and to evaluate whether these HER2 mutations are recurrent, we performed digital PCR with the same tumour tissues and CancerSCAN™ from archival breast tumour tissues. Our results suggest that there may be a patient population that receives little of no benefit from HER2-targeted therapies even though they have HER2-overexpressing BCs. HER2 mutations may be a main reason for this primary resistance to HER2-directed therapies.

Several mechanisms are thought to be responsible for resistance to HER2-targeted therapies. Several mechanisms of resistance to both trastuzumab and lapatinib have been identified in preclinical studies [17–23]. However, few of these have been validated prospectively in the clinic [6, 24, 25]. Unfortunately, the identification of a robust clinical or molecular predictor of trastuzumab benefit, including HER2 itself, has proven challenging [26–29]. There are no reports that HER2 somatic mutations play a role in primary resistance in HER2-amplified BCs, especially for patients with heavily pretreated disease, and there is insufficient scientific evidence to support this rationale.

Our results suggest that HER2 mutations may be useful as a predictive marker to identify which patients will not benefit from HER2-directed therapy. Emerging clinical data suggest that combinations of therapies targeting the HER2- signaling network at multiple points early in the natural history of HER2-positive breast cancer can abrogate drug resistance. For this reason, double-blockades may be regarded as an alternative for overcoming resistance. Obviously, patients with HER2 mutations will not be offered this therapeutic option because of the low activity of HER2 pathway in this population. Paradoxically, HER2 mutations are not considered to be the main driver of genetic alterations to override tumour aggressiveness, unlike in other malignancies, such as NSCLC and colorectal carcinomas [30–34]. HER2 somatic mutations have been shown recently to drive tumorigenesis in HER2-negative breast cancers [13]. We found a HER2 mutation in one HER2-negative patient (1/36, 2.6%) in the same site found in a previous report [13]. However, the other HER2 mutations were found mainly in patients with HER2-amplification (Table 2, Figure 3). Additionally, most of these mutations were recurrent mutations, although they only occurred in low frequencies. This result may derive from the deep targeted sequencing of CancerSCAN™, which may explain why deep targeted sequencing is needed for all exon sites as well as hot spots.

What remains a challenge is determining the precise resistance mechanism(s) in this particular type of patient. Answering this question will lead to the development of individualized and effective therapies for refractory MBC. This will require commitment to in-depth functional studies and molecular analysis of the tumors. Alternatively, the increasing use of preoperative therapy should provide a clinical research platform for the prediction of the response to combinations of anti-HER2 agents with cytotoxic chemotherapy to stratify patients for the following treatment(s) to improve therapeutic outcomes for patients with refractory HER2-positive BC. These residual cancers may be interrogated with open-ended molecular approaches to select those patients who might need to avoid conventional HER2-directed therapies.

In conclusion, HER2 mutations in patients with refractory BC may help explain the resistance to conventional HER2-directed therapies in HER2-positive BC.

## MATERIALS AND METHODS

### Patients

We conducted a prospective study at the Samsung Medical Center (SMC) from March 2013 to November 2014. The patients with breast cancer who progressed after or were refractory to conventional treatments, including endocrine therapies or chemotherapies such as anthracycline- and taxane- containing regimens, were recruited into this trial. Patients with HER2-positive BC had received at least two HER2-targeted therapies for 2 years before they enrolled in this trial.

### Genomic DNA extraction and quality measurement

Genomic DNA was extracted from fresh frozen tumour tissue and matched normal blood specimens using a QIAamp DNA Mini Kit (Qiagen, Valencia, CA, USA). Genomic DNA quality and quantity were analyzed using a NanoDrop 8000 UV-Vis spectrometer (Thermo Scientific Inc., Willington, DE, USA), Qubit 2.0 Fluorometer (Life Technologies Inc., Grand Island, NY, USA), and 2200 TapeStation instrument (Agilent Technologies, Santa Clara, CA, USA).

### Sequencing using a customized cancer panel (CancerSCAN™)

Genomic DNA (250 ng) from each tissue was sheared in a Covaris S220 ultrasonicator (Covaris, Woburn MA, USA) and used for the construction of a library using CancerSCAN™ probes and a SureSelect XT reagent kit, HSQ (Agilent Technologies) according to the manufacturer's protocol. This panel is designed to enrich exons of 83 genes (Supplemental Table 1), covering 366.2kb of the human genome. After enriched exome libraries were multiplexed, the libraries were sequenced on a HiSeq 2500 sequencing platform (Illumina). Briefly, a paired-end DNA sequencing library was prepared through gDNA shearing, end-repair, A-tailing, paired-end adaptor ligation, and amplification. After hybridization of the library with bait sequences for 27 hours, the captured library was purified and amplified with an index barcode tag, and the library quality and quantity were assessed. Sequencing of the exome library was performed using the 100 bp paired-end mode of the TruSeq Rapid PE Cluster Kit and TruSeq Rapid SBS Kit (Illumina).

### Variants detection using the customized cancer panel (CancerSCAN™)

Sequence reads were mapped to the human genome (hg19) using Burrows-Wheeler Aligner (BWA) [35]. Duplicate read removal was performed using Picard and SAMtools [36]. Local alignment was optimized using the Genome Analysis Toolkit (GATK) [37]. Variant calling was done only in regions targeted in CancerSCAN™. To detect single nucleotide variants, we integrated the results of three kinds of variant caller, which increased the sensitivity [38–40]. We used Pindel to detect indels [41]. Copy number variations were calculated for targeted regions by dividing the read depth per exon by the estimated normal reads per exon using an in-house reference. Among the variants obtained from the tumour tissue, germ-line events were filtered using the results of the matched blood sample.

### Gene expression and pathway analysis

FASTQ files from RNA sequencing of the SMC's sample from MBC patients were mapped to the human genome reference (hg19) using the bow-tie method [42]. TopHat was used to generate read counts per gene [43]. We normalized each patient's gene expression data to publically available breast cancer data. Gene expression data from the TCGA BRCA project (tumour = 526, normal = 61) were used for normalization. The statistical algorithm COMBAT was applied to reduce the platform and batch effects on data analysis [44]. After reducing the batch effect, we standardize each patients' gene expression value using the mean and standard deviation for gene expression obtained from normal breast tissue. An individualized pathway alteration score was obtained using the IPAS method [45].

### Bioinformatic analysis

All statistical analyses were performed by the Biostatistics and Clinical Epidemiology Center in our institute. We implemented the method found in the R “compound.Cox” package.

#### REMARK guidelines

In reporting our study, we have adhered to the guidelines of the important 2005 methodological paper entitled “Reporting recommendations for tumor marker prognostic studies (REMARK guidelines)” [46, 47].

### Digital PCR (polymerase chain reaction)

Digital PCR was performed using a QuantStudio 3D Digital PCR System platform comprising a Gene Amp 9700 PCR machine including a chip adapter kit, an automatic chip loader, and the QuantStudio 3D Instrument (Life Technologies, Inc.). The assay IDs of the primers and TaqMan probes are listed in Supplemental Table 5. We prepared 18 reaction mixtures containing 9 ul of two-fold QuantStudio 3D Digital PCR Master Mix (Life Technologies, Inc.), 0.9 ul of 20-fold TaqMan Assay by Design primer-probe mix, 2 ul diluted gDNA (100 ng/l), and 6.1 ul nuclease-free water (Biosesang, Korea). We loaded 14.5 ul of the reaction mixture onto a QuantStudio 3D Digital PCR 20K Chip (Life Technologies, Inc.) using an automatic chip loader according to the manufactures' instructions. Loaded chips underwent amplification in the Gene Amp 9700 PCR System under the following conditions: 96°C for 10 min, 42 cycles at 60°C for 2 min and at 98°C for 30 s, followed by a final extension step at 60°C for 2 min. After amplification, the chips were imaged on the QuantStudio 3D Instrument, which assesses raw data and calculates the estimated concentration of the nucleic acid sequence targeted by the FAM and VIC dye-labelled probes according to the Poisson distribution. The resulting data are reported in copies/ul along with the results of the quality assessment. For deeper analysis of the obtained chip data, QuantStudio 3D Analysis Suite Cloud Software (Life Technologies, Inc.) was used for relative and quantitative data analysis.

## SUPPLEMENTARY TABLES


