# Supplementary material for: Role of HER2 mutations in refractory metastatic breast cancers: targeted sequencing results in patients with refractory breast cancer
Source: Oncotarget. 2015 Sep 11;6(31):32027–38. doi: 10.18632/oncotarget.5184 (PMC4741657; doi:10.18632/oncotarget.5184)
Supplement: Supplementary file 1 [file oncotarget-06-32027-s001.pdf]

SUPPLEMENTARY TABLES

Supplementary Table S1: SMC CancerSCAN v1.1

	Specification			
**Target**	80 genes all exon + 5 genes translocation			
**Target Size**	366.231 Kb			
**Detection**	SNV, small Indel, CNV, Translocation			
**SNV and CNV**				
ABL1	BRCA2	FGFR2	KIT	PTCH1
AKT1	CDH1	FGFR3	KRAS	PTCH2
AKT2	CDK4	FLT3	MDM2	PTEN
AKT3	CDK6	GNA11	MET	PTPN11
ALK	CDKN2A	GNAQ	MLH1	RB1
APC	CSF1R	GNAS	MPL	RET
ARID1A	CTNNB1	HNF1A	MTOR	ROS1
ARID1B	DDR2	HRAS	NF1	SMAD4
ARID2	EGFR	IDH1	NOTCH1	SMARCB1
ATM	EPHB4	IDH2	NPM1	SMO
ATRX	ERBB2	IGF1R	NRAS	SRC
AURKA	ERBB3	ITK	NTRK1	STK11
AURKB	ERBB4	JAK1	PDGFRA	SYK
BCL2	EZH2	JAK2	PDGFRB	TOP1
BRAF	FBXW7	JAK3	PIK3CA	TP53
BRCA1	FGFR1	KDR	PIK3R1	VHL
**Translocation**				
ALK	EWSR1	ROS1	RET	TMPRSS2
TERT*				

Supplementary Table S2: Sequences of target amplification primers and TaqMan probes for digital PCR

Assay name	Forward Primer (5′- > 3′)	Reverse Primer (5′- > 3′)	Mutant allele probe (FAM-5′- > 3′)	Wild-type allele probe (VIC-5′ - > 3′)
S72A	ACCTACCTGCCCACCAATG	CGGGCCTCACCTGCA	AGCCTGGCCTTCC	CAGCCTGTCCTTCC
S413L	CTCTGCCTGCAGGTTACCTATAC	GAGGTCAGGCAGGCTGT	CGGCCATGCTAAGAT	CCGGCCATGCTGAGAT
Q692X	CAGAAGATCCGGAAGTACACGAT	GGGCTGGGAGGACTTCAC	CTCCGTTTCCTACAGCAGT	CCGTTTCCTGCAGCAGT
P420fs	GCAGGTTACCTATACATCTCAGCAT	CGGATTACTTGCAGGTTCTGGAA	CCTGCCGACCTCAG	CCTGCCTGACCTCAG
P562S	CCCCAGGGAGTATGTGAATGC	AACAGGTCACTGAGCCATTCTG	TGGCACGACAAACA	TGGCACGGCAAACA

Supplementary Table S3: Sequencing QC metrics of 36 MBC tumor samples

Sample Name	GC (%)	Q30 (%)	Duplication (%)	OnTarget (≥100x) (%)	OnBait (%)	Mean coverage
188	45	90	15.2	99.1	59.5	1024.·7
191	46	90.6	13.7	99.0	67.3	1079.8
200	45	90.2	13.0	99.1	63.4	919.5
209	46	90.8	11.9	99.1	67.9	947.4
226	46	89.4	14.1	99.2	60.6	962.5
230	47	89.8	10.0	98.9	66.0	973.6
235	45	91.4	21.9	99.0	61.3	785.8
240	46	90.4	9.6	98.9	64.2	964.6
244	45	91.7	12.1	98.8	67.1	896.6
245	46	91.9	10.9	98.7	66.1	927.7
250	44.5	91.7	12.5	98.8	68.4	910
255	45	90.3	7.8	98.7	58.7	837.9
256	47	90.8	21.6	98.8	67.1	815.7
257	47	90.2	15.9	98.8	65.7	840.4
259	46	91	34.9	99.1	63.4	860.7
262	45	91.6	27.0	98.6	71.1	973.7
263	46	91.1	15.8	99.1	61.4	961.4
265	46.5	91.4	10.7	98.9	65.0	1055.7
266	44	90.8	14.0	99.0	64.3	890.4
287	44.5	91.1	10.9	98.9	67.0	994
291	46	90.7	9.2	98.9	67.0	1046.7
306	45.5	90.8	30.1	99.0	65.9	785.3
173	46	93.1	11.5	99.2	65.9	1193.6
181	45.5	92.8	12.2	99.3	59.9	1296.6
228	45	92.7	14.7	99.0	65.1	973.5
251	46	92.7	11.9	98.9	67.1	1098.3
314	46	92.9	16.1	98.9	62.3	1006.8
321	45	91.8	16.7	99.2	60.9	1107.5
324	45	93	13.6	98.7	59.3	846.3
333	45	92.7	9.4	98.8	62.9	983.1
336	45	93.1	11.3	98.8	61.2	931.9
337	45	92.8	12.0	99.0	63.7	1018.3
349	46	92.6	20.6	99.6	62.4	778.8
375	45.5	92.8	17.5	99.5	53.7	750.9
410	46	92.8	21.3	99.6	63.9	779.3
370	46	92.4	26.2	99.6	57.0	667.1
Mean	45.6	91.6	15.5	99.0	63.7	941.3

Supplementary Table S4: Comparison of HER2 mutation with TCGA data

Sample ID	Protein Change	Mutation Type	Vaf (%)	CNV (copy no.)
226	P420fs	Frame Shift Del	0.2	AMP (14.0)
240	S413L	Missense Mutation	1.1	AMP (16.4)
256	S72A	Missense Mutation	0.8	AMP (25.6)
251	P562S	Missense Mutation	0.4	AMP (19.7)
321	Q692*	Nonsense Mutation	1.7	AMP (5.3)
266	**L755S**	Missense Mutation	19.0	Diploid (2)
TCGA-C8-A135	D769H	Missense Mutation	53.0	AMP (7.0)
TCGA-A2-A3XV	S310F	Missense Mutation	NA	AMP (3.6)
TCGA-EW-A1PD	H470Q	Missense Mutation	NA	Gain (2.7)
TCGA-A8-A06Z	G309A	Missense Mutation	NA	Gain (2.7)
TCGA-A8-A0A6	L755S	Missense Mutation	41.9	Gain (2.5)
TCGA-E9-A1R5	D769Y	Missense Mutation	NA	Gain (2.5)
TCGA-D8-A1XM	**L755S**	Missense Mutation	NA	Gain (2.5)
TCGA-A8-A0AB	**L755S**	Missense Mutation	50.5	Gain (2.4)
TCGA-BH-A18P	**L755S**	Missense Mutation	68.5	Gain (2.4)
TCGA-A2-A0T6	R678Q	Missense Mutation	23	Diploid (2)
TCGA-A2-A0T6	L755M	Missense Mutation	NA	Diploid (2)
TCGA-A2-A0T6	L755W	Missense Mutation	NA	Diploid (2)
TCGA-A8-A08Z	V842I	Missense Mutation	40.6	Diploid (2)
TCGA-AC-A3YI	L755S	Missense Mutation	NA	Diploid (2)
TCGA-AO-A128	V797A	Missense Mutation	NA	Diploid (2)
TCGA-BH-A0C1	V777L	Missense Mutation	36.5	Diploid (2)
TCGA-BH-A1FE	D769Y	Missense Mutation	NA	Diploid (2)
TCGA-C8-A274	I767M	Missense Mutation	NA	Diploid (2)
TCGA-OL-A5D6	V777L	Missense Mutation	NA	Diploid (2)
TCGA-AN-A046	T306M	Missense Mutation	NA	Hetloss (1.8)
TCGA-C8-A3M7	S305C	Missense Mutation	NA	Hetloss (1.7)

Supplementary Table S5: Gene expression differences of 43 genes in ERBB2/ERBB3 signaling pathway

Gene	Avg (ERBB2 amp/ wild, *n* = 7)	Avg (ERBB2 amp/ mut, *n* = 4)	Fold Change	T-statistic	*P*-value
GRB2	0.922	0.273	0.648	2.669	0.026
PIK3CB	0.366	−0.553	0.919	2.810	0.053
RAF1	0.380	−0.061	0.442	1.907	0.098
DOCK7	0.274	−0.243	0.517	1.843	0.098
MAPK1	−0.204	−0.873	0.670	1.803	0.130
ERBB3	1.673	0.988	0.685	1.640	0.148
MAP2K2	0.006	0.376	−0.370	−1.541	0.158
USP8	0.421	−0.030	0.451	1.559	0.192
BAD	0.762	1.231	−0.468	−1.280	0.236
RAC1	0.905	0.628	0.276	1.342	0.247
MAPK8	0.463	0.192	0.271	1.206	0.259
PPP3CB	1.038	0.678	0.359	1.274	0.270
PIK3CA	0.639	0.227	0.412	1.127	0.311
MAPK9	0.784	0.336	0·448	1.063	0.328
KRAS	0.525	0.301	0.224	1.024	0.332
PIK3CD	0.096	0.455	−0.359	−1.028	0.342
NRG2	−0.831	−0.317	−0.513	−0.997	0.348
CDC42	0.798	0.414	0.384	1.025	0.368
PIK3R3	1.401	1.094	0.307	0.955	0.375
SOS1	0.633	0.412	0.221	0.945	0.375
HRAS	0.318	0.703	-0.385	−0.903	0.397
PTPN11	0.382	0.048	0.334	0.900	0·405
PRKACA	0.510	0.295	0.215	0.827	0.447
NRG1	−1.032	−0.431	-0.600	−0.838	0.455
STAT3	1.181	0.921	0.259	0.718	0.500
ERBB2	3.249	3.527	-0.278	−0.697	0.509
CHRNA1	1.729	2.027	-0.298	−0.649	0.537
SRC	0.542	0.770	-0.228	-0.637	0.547
PIK3R1	2.348	1.995	0.353	0.558	0.602
JAK2	0.438	0.193	0.245	0.559	0.602
FOS	1.243	0.813	0.429	0.535	0.625
MAPK10	1.573	1.262	0.311	0.465	0.658
RNF41	1.059	0.865	0.194	0.479	0.658
JUN	1.272	1.450	-0.178	−0.358	0.733

(*Continued*)

Gene	Avg (ERBB2 amp/ wild, *n* = 7)	Avg (ERBB2 amp/ mut, *n* = 4)	Fold Change	T-statistic	*P*-value
NF2	−0.097	−0.213	0.116	0.336	0.752
NRAS	−1.006	−1.100	0.094	0.323	0.754
MAP2K1	−0.365	−0.447	0.083	0.290	0.787
AKT1	0.421	0.552	−0.131	−0.248	0·816
NFATC4	0.891	1.001	−0.110	−0.239	0.820
CHRNE	0.685	0.742	−0.057	−0.184	0.862
MAPK3	0.313	0.361	−0.048	−0.148	0.888
SHC1	0.331	0.348	−0.017	−0.058	0.956
PIK3R2	0.436	0.428	0.008	0.023	0.982

Among eleven ERBB2 amplified metastatic breast cancer patients, gene expression difference between ERBB2 wild type ($n = 7$) and ERBB2 mutated ($n = 4$) are compared
